# Identification of Docetaxel as a Potential Drug to Promote HDL Biogenesis

**DOI:** 10.3389/fphar.2021.679456

**Published:** 2021-05-21

**Authors:** Hong Y. Choi, Isabelle Ruel, Jacques Genest

**Affiliations:** Cardiovascular Research Laboratories, Research Institute of the McGill University Health Center, Montreal, QC, Canada

**Keywords:** desmocollin 1, docetaxel, cholesterol, atherosclerosis, HDL function

## Abstract

**Objective:** Our recent studies showed that desmocollin 1 (DSC1) binds to apoA-I in order to inhibit apoA-I-mediated high density lipoprotein (HDL) biogenesis in atherosclerotic plaques. To promote HDL biogenesis in the plaque, here we search for small molecules that block apoA-I-DSC1 interactions.

**Approach and Results:** We combined mutational and computational mapping methods to show that amino acid residues 442–539 in the mature DSC1 protein form an apoA-I binding site (AIBS). Using a crystal structure of the AIBS, we carried out virtual screening of 10 million small molecules to estimate their binding affinities to the AIBS, followed by the selection of 51 high-affinity binding molecules as potential inhibitors of apoA-I-DSC1 interactions. Among the 51, the chemotherapy drug docetaxel showed the highest potency in promoting apoA-I-mediated HDL biogenesis in primary human skin fibroblasts with the half-maximal effective concentration of 0.72 nM. *In silico* docking studies suggest that the taxane ring in docetaxel binds to the AIBS and that the carbon-13 sidechain of the taxane tightens/stabilizes the binding. The HDL biogenic effect of docetaxel was also observed in two predominant cell types in atherosclerosis, macrophages and smooth muscle cells. Importantly, docetaxel promoted HDL biogenesis at concentrations much lower than those required for inducing cytotoxicity.

**Conclusion:** Determination of the AIBS in DSC1 and AIBS structure-based virtual screening allowed us to identify docetaxel as a strong HDL biogenic agent. With the remarkable potency in promoting HDL biogenesis, a chemotherapy drug docetaxel may be repurposed to enhance atheroprotective HDL functions.

## Introduction

Although preventive and therapeutic efforts have made significant improvements in outcomes for atherosclerotic cardiovascular diseases (ASCVD), ASCVD remains a leading cause of morbidity and mortality worldwide (Roth et al., 2020). In the absence of a clinically useful high density lipoprotein (HDL)-directed therapy, recent studies measuring HDL activity such as HDL-mediated cholesterol efflux and HDL particle number suggest that HDL function, but not the circulating HDL-cholesterol level, is a clinically relevant biomarker and a therapeutic target for ASCVD (Rohatgi et al., 2014; Khera et al., 2017; Adorni et al., 2021). HDL particle formation and maturation occurring in the process of cellular cholesterol efflux is the best known mechanism of cholesterol removal from atherosclerotic plaques (Frambach et al., 2020). The HDL biogenic process is therefore considered to be the primary target for the development of HDL-directed therapies.

In order to study HDL biogenesis at the molecular level, we isolated plasma membrane (PM) micro-domains associated with the major structural and functional HDL protein, apolipoprotein A-I (apoA-I). Lipid and protein composition analysis of the micro-domain revealed a novel apoA-I binding PM micro-domain that is rich in cholesterol and contains desmocollin 1 (DSC1) (Choi et al., 2018). DSC1 is a member of the desmosomal cadherin family including desmogleins 1–4 and DSCs 1–3 in humans. Desmosomal cadherins are highly expressed in desmosome-forming tissues such as epithelial tissues and cardiac muscle. Although isoform-specific functions of four desmogleins and three DSCs remain to be elucidated, genetic and functional defects in all the desmosomal cadherins except DSC1 have been linked to human diseases including arrhythmogenic cardiomyopathy and various skin diseases (Kowalczyk and Green, 2013). In the absence of DSC1-linked human disease, mouse studies have shown that DSC1 may be dispensable for the formation of desmosomes (Chidgey et al., 2001; Cheng et al., 2004), suggesting that DSC1 may have unknown functions. Our studies have shown that DSC1 is abundantly expressed in macrophages and atherosclerotic lesions and that DSC1 in the micro-domain binds and prevents apoA-I from generating HDL particles and thus inhibition of apoA-I-DSC1 interactions promotes HDL biogenesis (Choi et al., 2018). The identification of DSC1 as a negative regulator of HDL biogenesis provided us with new insights into HDL and PM cholesterol regulatory mechanisms. Our current view of HDL biogenesis is as follows: ATP binding cassette A1 (ABCA1) transporter upregulated in cholesterol-laden cells creates a micro-domain in the PM for removal of excess cellular cholesterol by apoA-I, whereas DSC1 contained in another PM micro-domain interacts with apoA-I for the prevention of HDL biogenesis and conservation of PM cholesterol (Choi et al., 2018; Genest et al., 2018). The relative abundance of the two opposing PM micro-domains is a key factor determining HDL biogenesis, thus novel HDL-directed and anti-atherosclerotic therapies may be developed by inhibiting the interaction between apoA-I and DSC1.

To test our hypothesis, here we identified the apoA-I binding site in DSC1 and used the binding site structure to find small molecules that can dock into the site and thus function as apoA-I-DSC1 interaction inhibitors. A combination of high-throughput computational screening and biological validation methods allowed us to evaluate millions of small molecules prior to identifying docetaxel as a potent HDL biogenic agent. Our unbiased screening approach for molecules targeting apoA-I-DSC1 interactions has provided us opportunities to repurpose a chemotherapy drug docetaxel into HDL-directed therapies.

## Materials and Methods

### Antibodies

Anti-apoA-I antibody was purchased from Meridian Life Science (Cat. No. K45252G); anti-EGFP antibody from Clontech (Cat. No. 632569); anti-Actin antibody from Santa Cruz Biotechnology (Cat. No. sc-1616); anti-ABCA1 antibody from Millipore Sigma (Cat. No. MAB10005); anti-Desmocollin 1 antibody from Novus Biologicals (Cat. No. NBP1-88099); anti-alpha Tubulin antibody from Abcam (Cat. No. ab7291).

### Cell Culture

Primary human skin fibroblasts (HSFs) were obtained from a 3.0 mm punch biopsy of the forearm of a healthy control subject under protocol MEDB-2000-885 approved by the McGill University Health Center Ethics committee. This work conforms to the principles outlined in the Declaration of Helsinki, and written informed consent was obtained from the donor. These cells were cultured in DMEM (Wisent Bioproducts) supplemented with 10% (v/v) fetal bovine serum (Wisent Bioproducts), 1x penicillin/streptomycin (Wisent Bioproducts) and 1x nonessential amino acids (Wisent Bioproducts) in a humidified 37°C, 5% CO_2_ incubator. HEK293 cells (ATCC) were cultured in DMEM supplemented with 10% (v/v) fetal bovine serum and 1x penicillin/streptomycin in a humidified 37°C, 8.8% CO_2_ incubator. Human aortic smooth muscle cells (HASMCs) from Thermo Fisher Scientific Inc., were cultured in Medium 231 (Thermo Fisher) supplemented with 1x smooth muscle growth supplement (Thermo Fisher) and 1x penicillin/streptomycin in a humidified 37°C, 5% CO_2_ incubator. THP-1 monocytes (ATCC) were cultured in RPMI 1640 medium (Wisent Bioproducts) supplemented with 10% (v/v) fetal bovine serum and 1x penicillin/streptomycin in a humidified 37°C, 5% CO_2_ incubator. A high density culture enhances phorbol 12-myristage 13-acetate (PMA)-induced differentiation of THP-1 monocytes into macrophages (Aldo et al., 2013), so we cultured THP-1 monocytes at the density of 2.0 × 10^6^/ml with refreshing media every 2–3 days. The THP-1 monocytes were treated with 100 ng/ml of PMA (Sigma) for 3 days, washed with RPMI 1640 three times, and grown in the culture medium for 2 days in order to differentiate THP-1 monocytes into macrophages.

### Site-Directed Deletion Mutagenesis

Plasmid pDSC1b-EGFP encoding the full-length human DSC1b with C-terminal enhanced green fluorescent protein (EGFP) tag was constructed as described previously (Choi et al., 2018). The coding sequences of the fifth extracellular cadherin (EC5) domain of DSC1b were progressively deleted from the pDSC1b-EGFP in order to construct pDSC1b∆(447–466, 447–486, 447–506, 447–526, or 447–546)-EGFP. Numbers represent the positions of the amino acid residues, relative to the N-terminal amino acid of mature DSC1 protein. The series of 20 amino acid deletions were carried out using the In-Fusion HD Cloning Plus system (Clontech) that enables PCR mediated site-directed DNA mutagenesis. PCR primers used are as follows; one reverse primer (5′-TTC​TTT​GTC​AAT​TTG​AGG​TGC​GTG​ATC​GTT​G-3′) and five forward primers to delete amino acid residues 447–466 (5′-CAA​ATT​GAC​AAA​GAA​CCT​GAA​AAT​GGA​CCA​CCT​TTT​CAA​TTC-3′), amino acid residues 447–486 (5′-CAA​ATT​GAC​AAA​GAA​ATA​GAA​GAA​AAG​GAT​GGT​AAA​ACT​GC-3′), amino acid residues 447–506 (5′-CAA​ATT​GAC​AAA​GAA​TAT​TAT​TCT​GTG​CCT​ATT​CAA​ATA​AAA​GAC-3′), amino acid residues 447–526 (5′-CAA​ATT​GAC​AAA​GAA​ACA​GTG​AGA​GTA​TGT​GAC​TGT​TCA​AC-3′), or amino acid residues 447–546 (5′-CAA​ATT​GAC​AAA​GAA​AGA​GAC​GTT​AGA​CCA​AAT​GTA​ATA​CTT​GG-3′). PCR products were gel-purified prior to performing In-Fusion reaction according to the manufacturer’s protocol. Successful deletions of the target sequences were confirmed by sequencing the final constructs.

### Structure-Based Virtual Screening

A crystal structure of the human DSC1 ectodomain, 5IRY was retrieved from the RCSB protein data bank (https://www.rcsb.org) and prepared for molecular docking using Protein Preparation Wizard in Maestro (version 11.0). Maestro is the graphical user interface for all of Schrodinger’s computational programs including Protein Preparation Wizard, SiteMap, Grid Generation, LigPrep, Epik, and Glide (https://www.schrodinger.com/maestro). Protein Preparation Wizard was used to add missing hydrogen atoms, optimize hydrogen bonds, remove atomic clashes, and carry out other operations that were not included in the refinement process of X-ray crystal structure. Structural correctness and refinement made by the Wizard were essential for the accuracy of all downstream modeling simulations. SiteMap was used for identifying, evaluating, and visualizing possible ligand binding sites in the 5IRY. The sites were identified by calculating energetic and geometric properties suitable for protein-protein or protein-ligand interactions. The properties included the size of the site, the degrees of enclosure by the 5IRY and exposure to solvent, the degree to which a ligand might accept or donate hydrogen bonds, the hydrophobic and hydrophilic nature of the site and the balance between them, and the tightness between the site and site points assigned by SiteMap. A site-scoring function known as SiteScore within SiteMap computed these factors to prioritize possible binding sites. The second highest-scoring site was identified as a potential apoA-I binding site. Grid Generation was used to create a receptor grid that represents the geometrical shape and properties of the apoA-I binding site. To find ligands that favorably dock into the receptor grid, chemical structures of around 10 million compounds were obtained from Selleckchem, Enamine and ZINC compound databases. LigPrep was used to prepare all-atom three-dimensional molecular structures of the compounds. The LigPrep process consisted of a series of steps that added hydrogen atoms, eliminated unwanted molecules, neutralized charged groups, generated ionization states at physiological pH values, generated tautomers, specified chiralities, generated low-energy ring conformations, removed problematic structures, and optimized three-dimensional geometries. Many compounds can exchange protons with their environment, generating various ionization and tautomeric states, collectively called protonation states. Epik was used for accurate enumeration of compound protonation states in biological conditions. The compounds were subjected to Glide docking. Glide used a hierarchical series of filters to identify ligands that were energetically favorable in docking into the receptor grid. The filters tested the spatial fit of each ligand to the receptor grid, examined the complementary of ligand-receptor interactions, and evaluated the free energy of ligand-receptor interactions prior to scoring and rank-ordring ligands. The free energy values in favorable interactions are negative, thus high-affinity ligands are expected to have low Glide scores (Halgren et al., 2004; Friesner et al., 2006). Based on Glide scores and docking poses, 51 compounds were selected for biological testing.

### Low Density Lipoprotein Acetylation

LDL (*d* = 1.019–1.063 g/ml) was isolated by standard ultracentrifugation techniques from the pooled plasma of healthy volunteers (Chapman et al., 1981). Acetylation of LDL was performed as previously described (Basu et al., 1976; Kritharides et al., 1995). In brief, 1 ml of LDL in 150 mM NaCl was added to 1 ml of a saturated solution of sodium acetate with continuous stirring in an ice-water bath. Subsequently, 6 μl of acetic anhydride per milligram LDL protein was added in multiple small aliquots (2 μl) over a period of 1 h with continuous stirring, followed by stirring for an additional 30 min without acetic anhydride addition. The reaction solution was then dialyzed against 4 × 1 L PBS containing chloramphenicol (0.1 g/L) and Chelex-100 (1 g/L, Bio-Rad Laboratories) over 24 h at 4°C to remove excess saturated sodium acetate and acetic anhydride.

### Cholesterol Efflux Assays

Primary HSFs in 24-well plates were labeled with 0.2 μCi/ml of [^3^H]-cholesterol during the last 40% of growth to confluence. The cells were loaded with 30 μg/ml of unlabeled cholesterol for 24 h and then equilibrated for 24 h to upregulate ABCA1 expression. For the cholesterol loading, we used serum-free medium supplemented with 2 mg/ml of bovine serum albumin plus 30 μg/ml of cholesterol added from a 10 mg/ml stock solution in ethanol (Choi et al., 2003). The cells were then incubated with 5 μg/ml of apoA-I for 24 h to measure apoA-I-mediated cholesterol efflux. Indicated concentrations of chemical compounds were added during the equilibration and the apoA-I treatment periods. At the end of the incubation, media were collected and centrifuged at 2,000 g for 10 min to remove cell debris. The radioactivity in the medium and cell lysate was measured by liquid scintillation counting. Data were expressed as percentage of total (cell plus medium) [^3^H]-sterol appearing in the medium as previously described (Choi et al., 2003). Confluent HASMCs and differentiated THP-1 macrophages in 24-well plates were incubated with 50 μg/ml of acetylated LDL and 1 μCi/ml of [^3^H]-cholesterol for 24 h so as to load the cells with LDL cholesterol and to radiolabel cellular cholesterol pools (Yvan-Charvet et al., 2010). The cells were equilibrated for 24 h and then incubated with 5 μg/ml of apoA-I for 6 h to measure apoA-I-mediated cholesterol efflux. Indicated concentrations of docetaxel were added during the equilibration and the apoA-I treatment periods. At the end of the incubation, the radioactivity in the medium and cell lysate was determined as described above.

### Sulforhodamine B Cytotoxicity Assay

To evaluate docetaxel toxicity, the SRB assay was used (Skehan et al., 1990; Orellana and Kasinski, 2016). In brief, confluent HASMCs and differentiated THP-1 macrophages in 96-well plates were treated with indicated concentrations of docetaxel for 3 days. The cells were fixed with 50% of trichloroacetic acid for 2 h at 4°C, washed with water five times, air-dried overnight at room temperature, and stained for 30 min with 0.4% SRB solubilized in 0.1% acetic acid. The cells were rinsed five times with 1% acetic acid, air-dried overnight at room temperature, and dissolved in 10 mM Tris base solution. The optical density (OD) of the solution at 490 nm was measured to calculate the percentage of cell viability as follows: % cell viability = (OD_490_ sample/OD_490_ control) × 100.

### Statistical Analysis

Cholesterol efflux and SRB assay results were expressed as mean ± SD. One-way analysis of variance with Dunnett’s post-hoc correction was performed to calculate multiplicity adjusted *p* values using GraphPad Prism 6 software. A value of *p* < 0.05 is considered statistically significant.

## Results

### Mutational Analysis of the apoA-I Binding Site in DSC1

**FIGURE 1:**
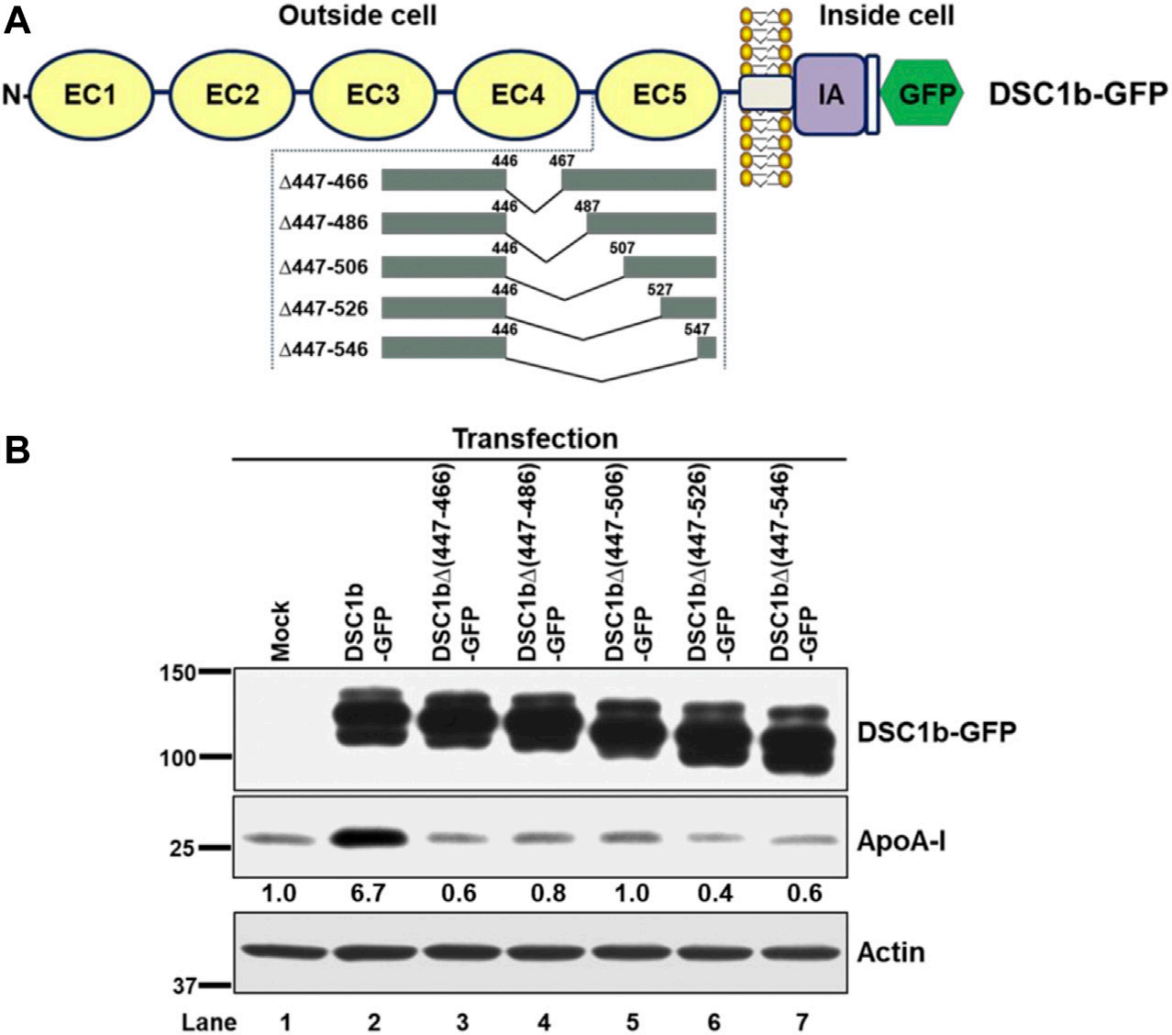
The domain structure of DSC1b protein and mutational analysis of apoA-I binding site. **(A)** DSC1b protein comprises five extracellular cadherin (EC1-EC5) domains, a single-pass transmembrane domain and an intracellular anchor (IA) domain. Green fluorescent protein (GFP) was fused to detect protein expression levels. The EC5 domain was progressively deleted to investigate if a particular part is responsible for apoA-I binding. The lengths and locations of the deleted parts are indicated by amino acid residue numbers **(B)** HEK293 cells were transfected with the constructs indicated. Two days after the transfection, the cells were maintained in Dulbecco’s modified Eagle’s medium supplemented with 1 mg/ml bovine serum albumin (DMEM/BSA) overnight to deplete serum-derived apoA-I. The cells were incubated with DMEM/BSA containing 5 μg/ml apoA-I for 1 h at 37°C. After extensive washing, the cells were lyzed to determine the levels of indicated proteins by immunoblotting. Numeric values shown below the apoA-I blot represent the densities of apoA-I bands normalized to actin and relative to mock-transfected cells (lane 1).

A newly identified apoA-I binding protein, DSC1 contains five tandemly repeated extracellular cadherin domains (EC1-EC5). Previous studies using cells expressing a series of truncated DSC1 proteins showed that the EC5 domain comprised of 80 amino acid residues (459–538) is essential for the interaction between apoA-I and DSC1 (Choi et al., 2018). Amino acid numbering starts at the amino-terminal amino acid of mature DSC1 protein here and throughout. To narrow down the apoA-I binding site in the EC5, plasmids encoding progressive EC5 deletion mutants were constructed (Figure 1A). HEK293 cells were transfected with the constructs to express the full-length DSC1b or a series of EC5 deletion mutants. The cells were incubated with apoA-I prior to determining the effects of progressive EC5 deletions on apoA-I binding (Figure 1B). Expression of the full-length DSC1b markedly increased apoA-I binding capacity (Figure 1B, lane 1 vs. 2), but the DSC1b-dependent apoA-I binding was not observed in cells expressing DSC1b∆447-466 (Figure 1B, lane 2 vs. 3). This complete abolishment of DSC1 effect on apoA-I binding suggested that the 20 residues (447–466) were crucial for apoA-I-DSC1 interactions.

### Computational Mapping of the apoA-I Binding Site in DSC1

To investigate if the 20 residues are involved in creating a protein binding site, we analyzed a crystal structure of the human DSC1 ectodomain (protein data bank ID = 5IRY) imported from the RCSB protein data bank (https://www.rcsb.org). Due to the limited resolution of crystallography, a protein crystal structure in its raw state is not suitable for molecular modeling. Common problems include missing atoms and incorrect bond orders, protonation states and charges, or orientations of chemical groups. To prepare the DSC1 crystal structure for use in molecular modeling, we used the Protein Preparation Wizard in the Schrodinger software graphical user interface called Maestro (version 11.0). The Wizard augmented DSC1 crystal data by fixing structural defects, removing unwanted molecules and optimizing DSC1 structure. The first step was to ensure the chemical correctness of DSC1 by correcting defective bond order assignments, adding missing hydrogens, creating zero-order bonds to metals, creating disulfide bonds, filling in missing side chains, and capping termini. In the second step of review and modification, dimeric DSC1 structure was reduced to monomer. Also, ionization or tautomeric states of co-crystalized heteroatom groups such as ions and cofactors were corrected. In the final refine step, hydrogen-bond assignment was optimized, water molecules with less than three hydrogen-bonds to non-waters were removed, and the corrected structure was minimized to alleviate any significant steric clashes. The finalized DSC1 structure for molecular modeling is shown in Supplementary Figure S1.

**FIGURE 2:**
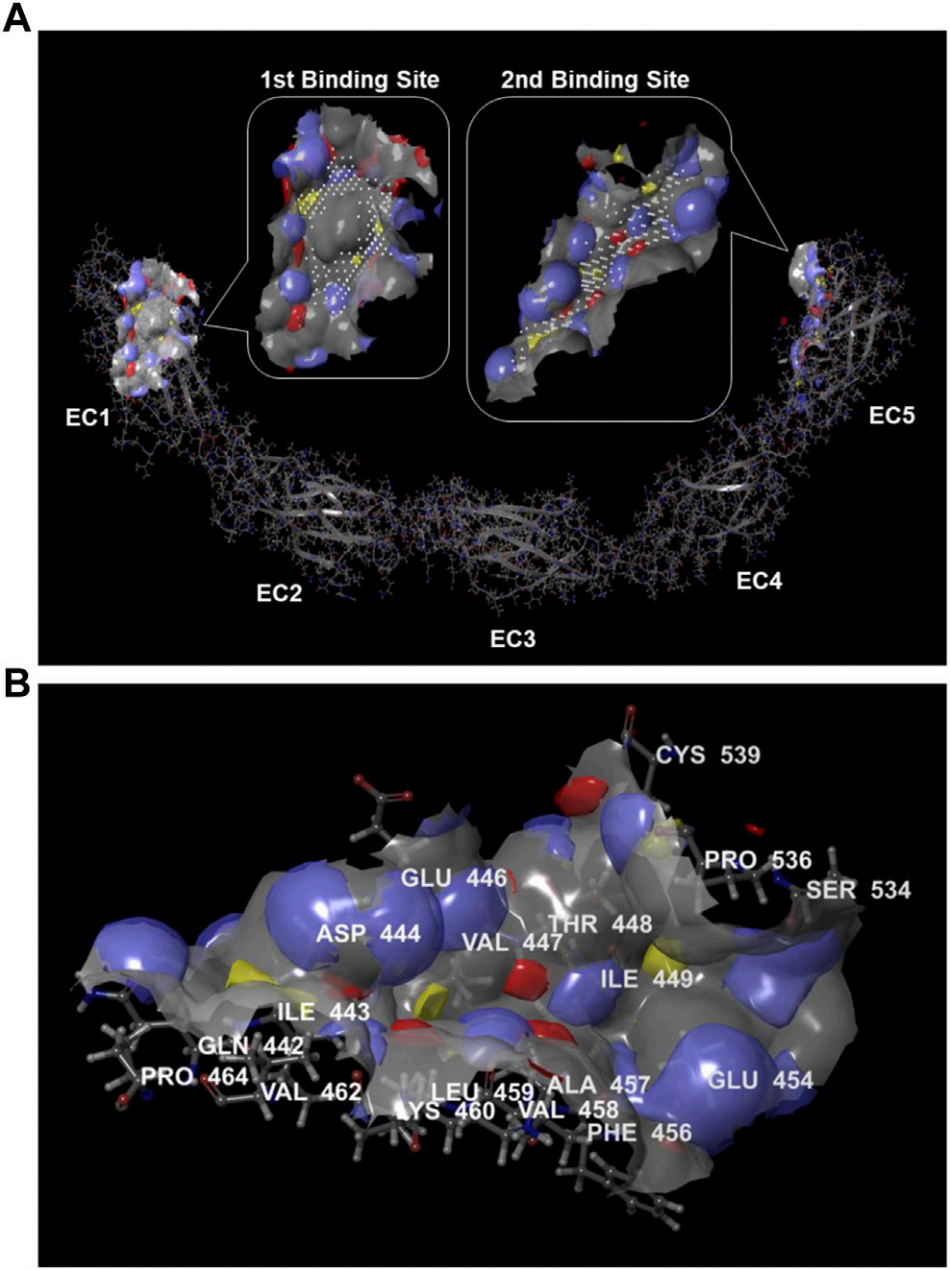
Protein binding sites in DSC1. **(A)** Potential protein binding sites in DSC1 were calculated by the SiteMap algorithm. The first-ranked binding site was in the EC1 domain and the second-ranked one was in the EC5 plus a part of the region between EC4 and EC5 domain. Color scheme for the binding sites displayed in rounded rectangular callouts: hydrogen-bond acceptor regions in blue, hydrogen-bond donor regions in red, hydrophobic regions in yellow, binding site points in white, and the binding site surface in gray **(B)** A display of the second highest-scoring binding site identified by the SiteMap. Amino acid residues located within a radius of 3 Å from the binding site are labeled. Hydrogen-bond acceptor regions are colored blue, hydrogen-bond donor regions in red, hydrophobic regions in yellow, and the binding site surface in gray.

The presence of protein binding sites in the DSC1 was calculated by the SiteMap tool in Maestro. Binding sites identified by the SiteMap algorithm were represented as collections of site points at or near the surface of DSC1 that are contiguous or separated in solvent-exposed region by short gaps that could plausibly be spanned by ligand functionality. To visualize binding site features, a grid of points to identify potential hydrophobic and hydrophilic regions was used; the hydrophilic regions were further classified into hydrogen-bond donor and hydrogen-bond acceptor regions, and the binding site surface was contoured. Based on binding site properties such as size, functionality and extent of solvent exposure, an overall SiteScore that assesses a site’s propensity for ligand binding was calculated in order to rank possible binding sites. The highest-scoring binding site was found in the EC1 domain and the second one in the EC5 plus a part of the region between EC4 and EC5 domain (Figure 2A). Desmosomal cadherin proteins including DSC1 are known to bind through their EC1 domains in order to form homophilic or heterophilic dimers (Nie et al., 2011; Harrison et al., 2016), indicating that binding sites identified by the SiteMap are reliable. There is no known protein interacting with the second binding site, but interestingly amino acid residues comprising the binding site within a radius of 3 Å are largely coincided with the 20 residues (447–466) that are crucial for apoA-I-DSC1 interactions (Figure 2B). These results strongly suggest that apoA-I may bind to the second binding site and that small molecules being able to bind to the site may block apoA-I-DSC1 interactions.

### Structure-Based Virtual Screening of Small Molecules

In order to identify small molecules that inhibit apoA-I-DSC1 binding, the physical properties of the volume of the predicted apoA-I binding site were specified using the Receptor Grid Generation program in Maestro. The van der Waals radii of nonpolar DSC1 atoms were left unchanged by setting the scaling factor of van der Waals radius at 1.0; nonpolar was defined by the partial atomic charge less than 0.25. A grid area encompassing the binding site was calculated and enclosed by a box at the centroid of SiteMap points (Supplementary Figure S2). The receptor grid represents the active site of DSC1 and we searched for small molecule ligands that can dock into the active site.

Databases of commercially-available small molecules are freely downloadable, and we obtained structure data of around 10 million small molecules from Selleckchem (http://www.selleckchem.com), Enamine (http://www.enamine.net) and ZINC (http://zinc.docking.org) compound libraries. We used the grid-based ligand docking with energetics (Glide) program in Maestro to screen the libraries in search of potential ligands for the DSC1 grid. To achieve a good Glide docking performance, each ligand structure must be three-dimensional, have realistic bond lengths and bond angles, consist of a single molecule that has no covalent bond to the receptor, have all its hydrogens, and have an appropriate protonation state for physiological pH values. The preparation of ligand structures for Glide was done using the LigPrep program in Maestro. In cases of complex ligands, LigPrep produced multiple output structures for a single input structure by generating different protonation states, stereochemistry, tautomers, and ring conformations.

To calculate computational docking of the prepared ligands into the DSC1 receptor grid, Glide performed a systemic search of the conformational, orientational and positional space of each ligand docked in order to generate an accurate pose for each ligand-receptor complex. Ligand-receptor interactions such as hydrogen bonds and hydrophobic contacts are scored to estimate the free energy of ligand binding. Based on the binding free energies, ligands that favorably interact with the receptor are rank-ordered. To decrease penalties for close ligand-receptor contacts, the van der Waals radii of nonpolar ligand atoms were scaled by 0.8; nonpolar was defined by the partial atomic charge less than 0.15. The docking job was performed with the setting of docking ligands flexibly, penalizing amide C-N bonds that are not cis or trans conformation, and adding Epik ionization and tautomeric state penalties to docking scores. After performing virtual screening of the ligands with the standard-precision docking method, the top-ranked 10% of ligand poses were reanalyzed by the extra-precision docking method. Based on extra-precision docking scores and docking poses, we chose 51 favorable ligands for the active site of DSC1. The overall screening work-flow is simplified in Supplementary Figure S3.

### Identification of Three Biologically Effective Compounds

**FIGURE 3:**
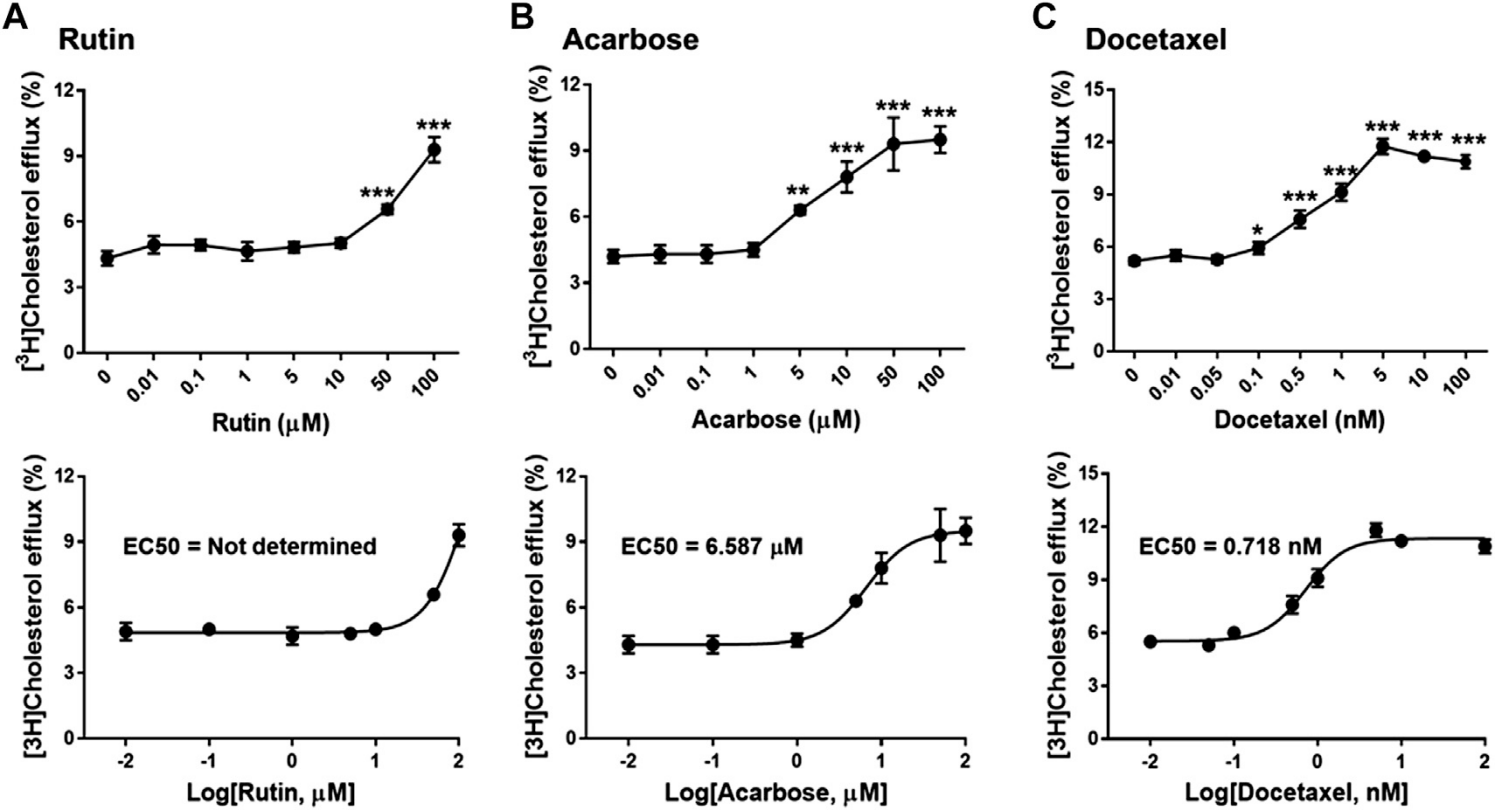
Rutin **(A)**, acarbose **(B)** and docetaxel **(C)** promote apoA-I-mediated cholesterol efflux. Primary HSFs were labeled with 0.2 μCi/ml of [^3^H]-cholesterol during growth, loaded with 30 μg/ml of unlabeled cholesterol for 24 h, equilibrated for 24 h, and treated with 5 μg/ml of apoA-I for 24 h to measure efflux of cellular cholesterol by apoA-I. The indicated concentrations of rutin, acarbose and docetaxel were added during the equilibration and apoA-I treatment period. Results are expressed as percentage of total (cell plus medium) [^3^H]-sterol appearing in the medium. Values are the mean ± SD of quadruplicate determinations. One-way analysis of variance with Dunnett’s post-hoc correction was performed to calculate multiplicity-adjusted *p* values. *p < 0.05; **p < 0.001; ***p < 0.0001 compared with the group treated with apoA-I alone.

To investigate biological effects of the 51 small molecule compounds on HDL biogenesis, cellular cholesterol efflux mediated by apoA-I was measured in the presence of the compounds in primary HSFs (Choi et al., 2003). Three compounds out of the 51 were particularly effective in promoting HDL biogenesis and their chemical structures and extra-precision docking scores are shown in Table 1. Dose-response curves for the three compounds showed that the most potent one was docetaxel having the half-maximal effective concentration (EC50) of 0.72 nM and that the second and third most potent ones were acarbose and rutin, respectively (Figure 3). Docetaxel has the highest Glide docking score (i.e., the lowest docking affinity) among the three compounds (Table 1), indicating that there is no exact correlation between docking score and biological activity. The effect of the three compounds on cholesterol efflux was not observed in the absence of apoA-I, suggesting that all the compounds work through the same mechanism involving apoA-I. As ABCA1 is required for apoA-I-mediated cholesterol efflux, these results suggest that the three compounds chosen as favorable ligands for the active site of DSC1 may promote ABCA1-dependent HDL biogenesis by inhibiting apoA-I-DSC1 interactions.

**TABLE 1:** Three chemical compounds that dock into the active site of DSC1 and promote HDL biogenesis.

Chemical structure	Name	Formula	Mol. weight	CAS Number	Docking score
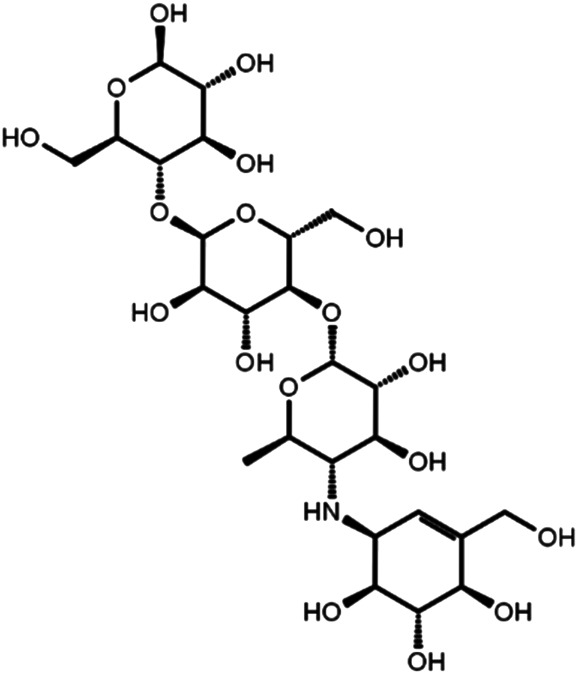	Acarbose	C25H43NO18	645.6	56180-94-0	−10.29
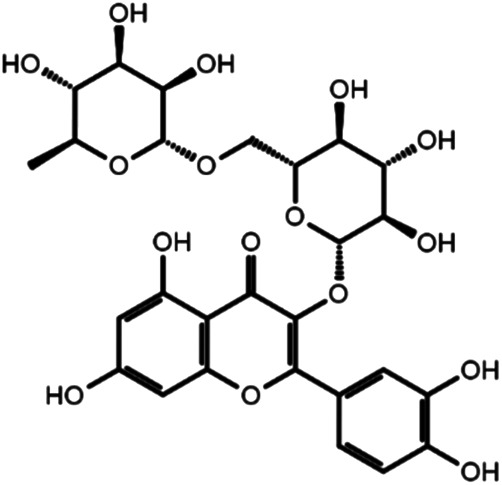	Rutin	C27H30O16	610.5	153-18-4	−7.88
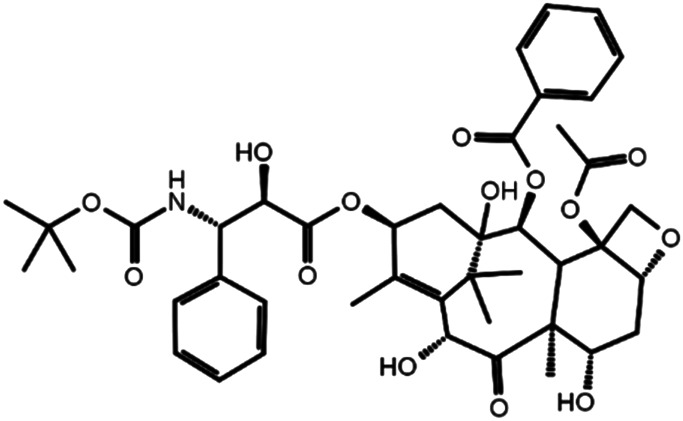	Docetaxel	C43H53NO14	807.9	114977-28-5	−7.07

### Characteristics of Biologically Effective Compounds

**FIGURE 4:**
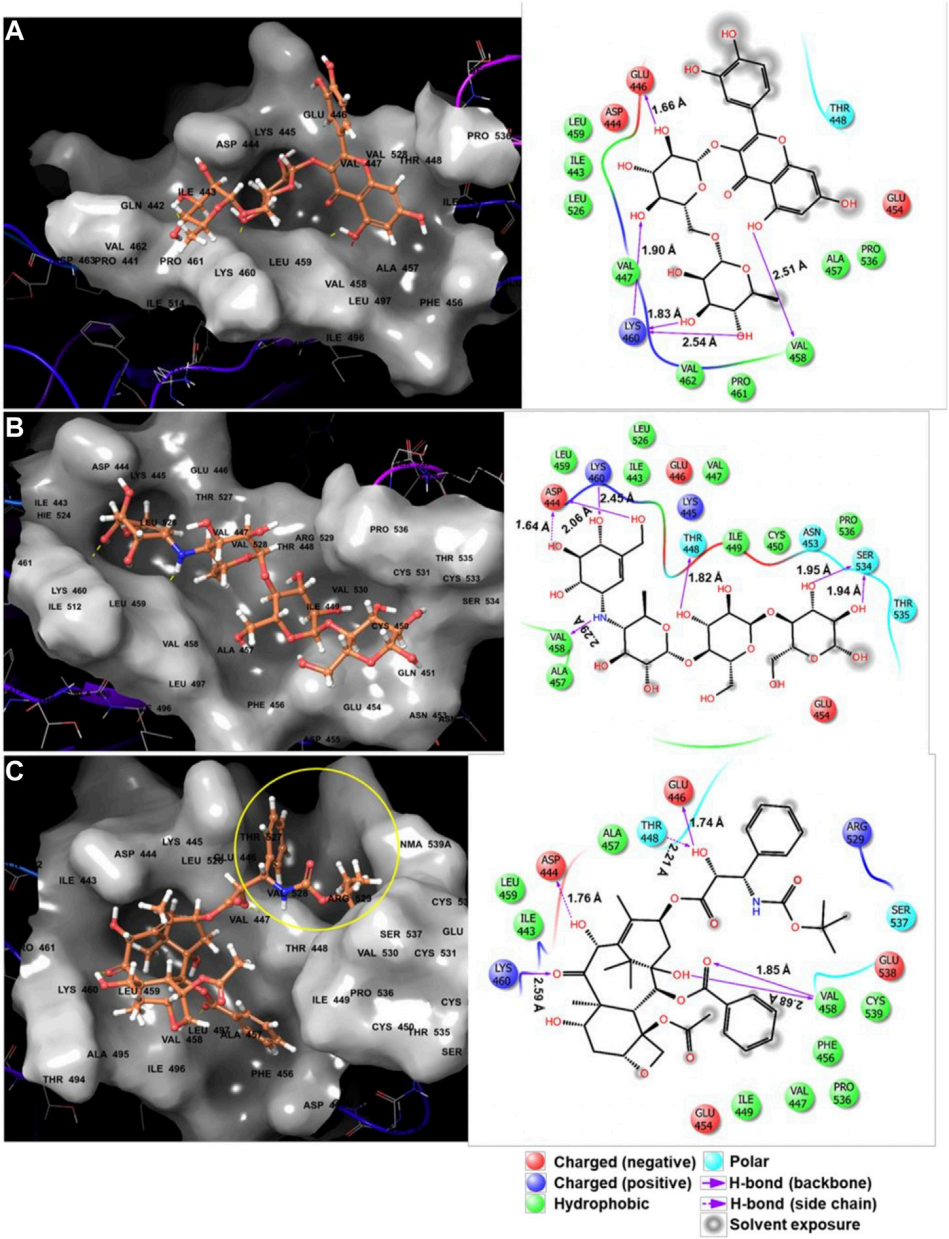
Predicted binding poses and interaction diagrams of rutin **(A)**, acarbose **(B)** and docetaxel **(C)** in the active site of DSC1.

The active site of DSC1 consists of a few shallow hydrophobic pockets and many hydrogen-bonding regions (Figure 2B). All of the three effective compounds are rich in hydrogen-bonding donor or acceptor atoms (Table 1). These results suggest that hydrogen bonds are the most important interactions between the DSC1 active site and an effective compound. Among the three effective compounds, rutin showed the lowest potency in promoting HDL biogenesis (Figure 3A) and its Glide docking score was -7.89 (Table 1). Based on the DSC1 crystal structure 5IRY, rutin was simulated to form three strong hydrogen bonds with Glu446 (1.66 Å, the hydrogen bond distance) and Lys460 (1.90 and 1.83 Å), and two moderate hydrogen bonds with Lys460 (2.54 Å) and Val458 (2.51 Å), as displayed in Figure 4A. Acarbose having the lowest Glide docking score (i.e., the highest docking affinity) among the three compounds (Table 1) promoted HDL biogenesis in primary HSFs with the EC50 of 6.59 μM (Figure 3B). As displayed in Figure 4B, acarbose was simulated to form six strong hydrogen bonds with Asp444 (1.64 and 2.06 Å), Thr448 (1.82 Å), Val458 (2.29 Å) and Ser534 (1.94 and 1.95 Å), and one moderate hydrogen bond with Lys460 (2.45 Å). Docetaxel showing the highest potency in promoting HDL biogenesis in primary HSFs with the EC50 of 0.72 nM (Figure 3C), despite the highest Glide docking score (Table 1), was simulated to form four strong hydrogen bonds with Asp444 (1.76 Å), Glu446 (1.74 Å), Thr448 (2.21 Å) and Val458 (1.85 Å), and two moderate hydrogen bonds with Lys460 (2.59 Å) and Val458 (2.68 Å), as displayed in Figure 4C. All of the three effective compounds had hydrogen bond interactions with Val458 and Lys460, and two of the three with Asp444, Glu446 and Thr448 (Figure 4). These five DSC1 residues may also play roles in interacting with apoA-I. Three residues in the DSC1 active site were predicted to form hydrogen bonds with rutin, while five residues with acarbose and docetaxel (Figure 4), suggesting that the potency of a compound may depend on the number and the position of residues with which the compound is able to form hydrogen bonds.

Among nine hydrophobic residues displayed in each of the three ligand interaction diagrams, six residues (Ile443, Val447, Ala457, Val458, Leu459 and Pro536) were common for all three of the effective compounds (Figure 4), suggesting that hydrophobic interactions may also contribute to enhancing compound activities.

The remarkable potency of docetaxel may be attributed to its unique chemical and conformational properties. Docetaxel is composed of a taxane ring with an ester sidechain attached at carbon (C)-13 of the taxane ring (Supplementary Figure S4) (Mastropaolo et al., 1995). The taxane ring is predicted to dock into the apoA-I binding site through hydrogen bond and hydrophobic interactions. Acarbose and rutin are also predicted to dock to the same binding site (Figure 4), therefore the taxane ring alone may not be sufficient to explain the potency of docetaxel. One of major distinctions of docetaxel from rutin and acarbose is that docetaxel is predicted to interact with additional binding cavities indicated by a yellow circle in Figure 4C. The C-13 sidechain of docetaxel contains the phenyl ring and the *tert*-butoxycarbonyl group. The two chemical groups are simulated to interact with the additional binding cavities that were not included in the apoA-I binding site seen in Figure 2B. A hydroxyl group positioned immediately before the phenyl ring forms two strong hydrogen bonds with Glu446 (1.74 Å) and Thr448 (2.21 Å) as displayed in Figure 4C, which may lead or stabilize the interactions between the C-13 sidechain and the cavities. The combination of the taxane ring-attributed docking into the apoA-I binding site and the C-13 side chain-attributed tighter-binding may allow docetaxel to exert the outstanding potency in promoting HDL biogenesis (Figure 3; note the change in the *X* axis scale, now in nM).

Considering that virtual high-throughput screening is a useful method to find potential lead compounds for drug discovery but that the method alone is often not sufficient to identify compounds working at low nanomolar concentrations, the identification of docetaxel is very fortunate and the potency makes it possible to test docetaxel as a drug candidate without going through further optimization processes.

### Docetaxel Effects on Atherosclerosis-Relevant Cells

**FIGURE 5:**
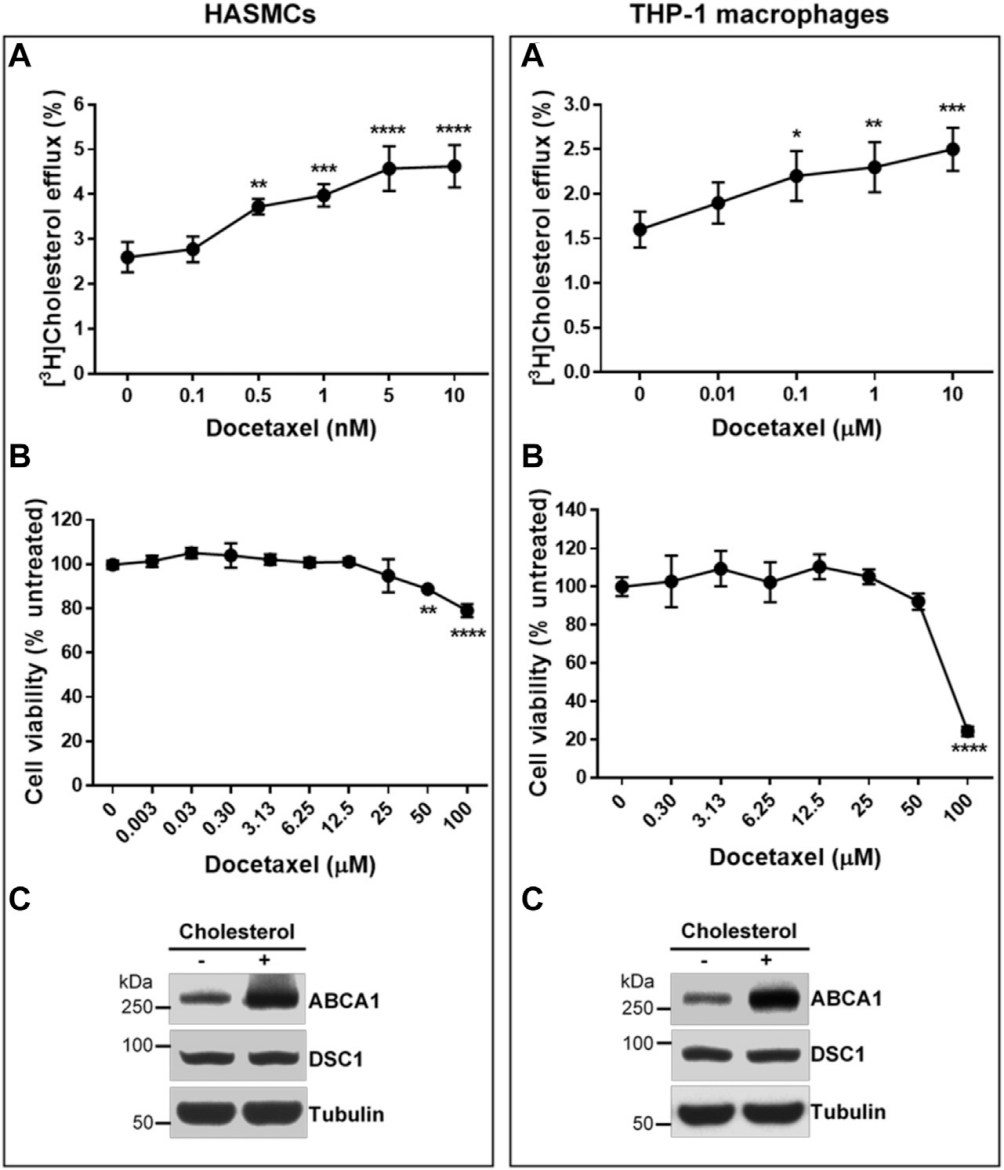
Docetaxel at non-cytotoxic concentrations promotes apoA-I-mediated cholesterol efflux in HASMCs and THP-1 macrophages. **(A)** Confluent HASMCs and differentiated THP-1 macrophages were incubated with 50 μg/ml of acetylated LDL and 1 μCi/ml of [^3^H]-cholesterol for 24 h, equilibrated for 24 h, and treated with 5 μg/ml of apoA-I for 6 h to measure efflux of cellular cholesterol by apoA-I. The indicated concentrations of docetaxel were added during the equilibration and apoA-I treatment period. Results are expressed as percentage of total (cell plus medium) [^3^H]-sterol appearing in the medium. Values are the mean ± SD of quadruplicate determinations. One-way analysis of variance with Dunnett’s post-hoc correction was performed to calculate multiplicity-adjusted *p* values. *p < 0.05; **p < 0.01; ***p < 0.001; ****p < 0.0001 compared with the group untreated with docetaxel in each cell type **(B)** Confluent HASMCs and differentiated THP-1 macrophages were treated with indicated concentrations of docetaxel for 3 days, fixed with 50% trichloroacetic acid, and stained with 0.4% SRB dye. The dye extracted from the cells were quantified by measuring its optical density (OD) at 490 nm. The amount of dye is proportional to the cell mass, thus the effect of docetaxel on cell viability was determined as follows: % cell viability = (OD_490_ treated/OD_490_ untreated) × 100. Values are the mean ± SD of triplicate determinations. Statistical analysis was performed as described above **(C)** Confluent HASMCs and differentiated THP-1 macrophages were incubated with or without 30 μg/ml of cholesterol for 24 h, equilibrated for 24 h, and lyzed to determine protein expression levels by immunoblotting.

Two major cell types involved in the initiation and progression of atherosclerosis are macrophages and smooth muscle cells (Allahverdian et al., 2012; Allahverdian et al., 2014). Unregulated uptake of modified LDL particles by these cells in the arterial wall leads to the formation of atherosclerotic macrophage/smooth muscle foam cells. HDL biogenesis in the foam cells is believed to be the chief anti-atherogenic effect of HDL. To test if docetaxel promotes HDL biogenesis in the atherosclerosis-relevant cells, THP-1 macrophages and human aortic smooth muscle cells (HASMCs) were loaded with acetylated LDL prior to measuring apoA-I-mediated cholesterol efflux. Docetaxel led to a concentration-dependent promotion of HDL biogenesis in both THP-1 macrophages and HASMCs. The EC50 of the docetaxel effect on HASMCs was 0.35 nM, while the effect on THP-1 macrophages did not reach a plateau in the concentration range from 0.01–10 μM (Figure 5A). In view of macrophages as specialized cells that are able to uptake and store substantial amounts of cholesterol, the necessity of high docetaxel doses for THP-1 macrophages suggests that macrophages may have larger storage pools of cholesterol and smaller efflux-available pools of cholesterol, compared to other cell types. The United States Food and Drug Administration (FDA) has approved docetaxel as a chemotherapy drug, and we evaluated the cytotoxic effect of docetaxel on the cells using the SRB assay (Skehan et al., 1990; Orellana and Kasinski, 2016). Cytotoxicity was not observed until 25 μM of docetaxel in HASMCs and 50 μM in THP-1 macrophages (Figure 5B). The expression levels of an HDL biogenic factor ABCA1 were upregulated in HASMCs and THP-1 macrophages in response to cholesterol loading, while DSC1 expression levels remained unchanged (Figure 5C). Combined, these results suggest that non-cytotoxic concentrations of docetaxel may be used to promote HDL biogenesis in DSC1-expressing macrophages and smooth muscle cells in the atherosclerotic plaque.

## Discussion

Excess cholesterol accumulated in peripheral tissues is transported to the liver for disposal or recycling of cholesterol, known as reverse cholesterol transport (Rosenson et al., 2012; Plummer et al., 2021). HDL particles generated in the process of removing cellular cholesterol initiate the reverse cholesterol transport pathway, therefore HDL biogenesis in the atherosclerotic plaque is thought to be a biomarker of atheroprotective HDL function (Choi et al., 2017). The identification of a novel apoA-I binding protein DSC1 as a negative regulator of HDL biogenesis suggests that apoA-I-DSC1 interactions may contribute to apoA-I retention in the atherosclerotic plaque and thus low HDL levels in ASCVD (Choi et al., 2018; Schwertani et al., 2018). In search of apoA-I-DSC1 interaction inhibitors, here we screened around 10 million small molecules and show that rutin, acarbose and docetaxel have the potential to be developed as DSC1-targeting HDL biogenic agents. Intriguingly, all of the three small molecules have already been approved for use as drugs by the FDA.

Rutin is a flavonol glycoside found in a variety of plants including citrus fruit, buckwheat, tobacco, forsythia, hydrangea and viola. A wide range of pharmacological effects of rutin includes atheroprotective actions such as anti-oxidative, anti-ischemic and anti-platelet aggregation effects (Ortolani et al., 1995; Navarro-Nunez et al., 2008). Diabetic patients supplemented with rutin increase HDL-cholesterol (HDL-C) while decrease LDL-cholesterol (Sattanathan et al., 2010). These data suggest that rutin may improve cardiovascular conditions. In view of the weak activity of rutin in promoting HDL biogenesis (Figure 3A), however, it can be quite challenging to attain sufficient concentrations of rutin in the circulation for the achievement of atheroprotective HDL biogenic effects.

Acarbose is a pseudo-carbohydrate that binds and inhibits pancreatic alpha-amylase and intestinal alpha-glucosidase activities (Wehmeier and Piepersberg, 2004; Standl and Schnell, 2012). Delayed digestion and absorption of dietary carbohydrates by acarbose reduce postprandial blood glucose levels, which in turn decreases hepatic lipogenesis and circulating triglyceride (TG) levels. Like acarbose, other glucose-lowering drugs also decrease TG levels in the circulation (Monami et al., 2012), which is in line with the tight link between glucose and TG metabolism. Unlike the uniform effect on TG levels, glucose-lowering drugs have variable effects on circulating HDL-C levels: increased by acarbose, but decreased by sulfonylureas, while not significantly affected by dipeptidyl peptidase 4 inhibitors (Monami et al., 2012). The HDL-C raising effect of acarbose was also observed in non-diabetic conditions (Zhang et al., 2014; Jalalvand et al., 2015). These data suggest that acarbose-mediated increase in HDL-C may be not associated with glucose metabolism but resulted from inhibiting apoA-I-DSC1 interactions. Owing to the close interaction between glucose and lipid metabolism, lipid abnormalities are prevalent in diabetic patients. Dyslipidemia is one of the major risk factors for ASCVD in diabetes mellitus and the dominant abnormality of diabetic dyslipidemia is hypertriglyceridemia and low HDL-C (Wu and Parhofer, 2014; Parhofer, 2015). As acarbose decreases TG and increases HDL-C levels, acarbose may be effective for the management of diabetic dyslipidemia. A problem is that the bioavailability of acarbose is very low: acarbose is metabolized by intestinal bacteria and digestive enzymes within the gastrointestinal tract, and less than 2% of an oral does is absorbed into the circulation (McIver and Tripp, 2018). When acarbose was given intravenously, 89% of the dose was recovered in the urine within 48 h (http://dailymed.nlm.nih.gov/dailymed/lookup.cfm?setid=6c2db888-775c-4baf-a1b4-1cfa63b83357). The low bioavailability due to poor absorption and rapid clearance may be a major limiting factor to use acarbose as an HDL biogenic drug. In support of the concept, the STOP-Noninsulin-Dependent Diabetes Mellitus trial showed that 100 mg of oral acarbose three times daily reduced cardiovascular events by 49% (hazard ratio 0.51; 95% confidence interval 0.28–0.95; *p* = 0.03) (Chiasson et al., 2003), whereas the Acarbose Cardiovascular Evaluation trial showed that 50 mg of oral acarbose three times daily did not reduce the risk of major adverse cardiovascular events (hazard ratio 0.98; 95% confidence interval 0.86–1.11; *p* = 0.73) (Holman et al., 2017). The FDA-approved maximum daily does is 100 mg three times a day, but the dose should not exceed 50 mg three times a day when the patient weighs less than 60 kg (McIver and Tripp, 2018), due to gastrointestinal side-effects such as flatulence, diarrhea, and rectal bleeding. These studies suggest that cardiovascular benefits of acarbose may depend on its concentrations in the circulation and that development of acarbose delivery systems to enhance bioavailability and reduce side-effects may be required for the expansion of acarbose’s therapeutic potential in the treatment of cardiovascular disease.

Docetaxel, a semi-synthetic analogue of paclitaxel (Taxol), is an anti-mitotic chemotherapy drug. By binding and stabilizing microtubules, docetaxel prevents mitotic spindle assembly and thus blocks cell cycle progression in late G2-M phase (Yvon et al., 1999; Morse et al., 2005). Chemotherapy agents affect HDL metabolism in varying ways: docetaxel increases HDL and decrease TG levels in the serum of mice bearing Ehrlich tumor (Alkhatib et al., 2017), but doxorubicin decreases HDL biogenesis by reducing the expression of ABCA1 and apoA-I, while cyclophosphamide and paclitaxel show no significant effect on HDL biogenesis (Sharma et al., 2016). Although there is no study investigating the effect of docetaxel monotherapy on lipid metabolism in humans, our discovery of docetaxel as a potent promoter of HDL biogenesis (Figures 3, 5) suggests that docetaxel may be repurposed for the treatment of ASCVD. In favor of the concept, another atheroprotective action of docetaxel has been reported: by suppressing the activation of platelet-derived growth factor (PDGF) beta receptor, docetaxel inhibits proliferation of vascular smooth muscle cells (VSMCs) (Park et al., 2011). Aberrant proliferation of VSMCs in the arterial wall is a crucial event in the development of atherosclerosis and restenosis following angioplasty or bypass surgery (Dzau et al., 2002; Doran et al., 2008; Marx et al., 2011). As the PDGF signaling pathway plays a prominent role in the proliferation and migration of VSMCs in atherosclerotic lesions (Boucher et al., 2003; Raines, 2004; Millette et al., 2006), the anti-proliferative effect of docetaxel provides further impetus for the repurposing of docetaxel as an anti-atherogenic drug. Both anti-proliferative (Park et al., 2011) and HDL biogenic (Figure 5) effects of docetaxel were observed at non-cytotoxic concentrations, suggesting that cytotoxicity issues with docetaxel may be circumvented as well.

In chemotherapy practice, docetaxel is usually administered intravenously to increase dose precision, but oral administration of docetaxel is feasible. Its oral bioavailability has been found to be 8% on its own and the bioavailability increased to 90% when co-administered with cyclosporine (Malingre et al., 2001). The concentration-time profile of docetaxel follows a three-compartment pharmacokinetic model with half-lives for the alpha, beta and gamma phases of 4.5 min, 38.3 min, and 12.2 h, respectively (Clarke and Rivory, 1999). The alpha half-life, the rate of decline in plasma concentrations represents the distribution of docetaxel to peripheral compartments from the circulation. The beta half-life of 38.3 min and the relatively slow gamma half-life of 12.2 h represent a slow elimination of docetaxel from peripheral compartments. Docetaxel is uptaken by a wide range of tissues and is minimally excreted by the kidneys (Clarke and Rivory, 1999). These pharmacokinetic properties are in favor of using docetaxel for the treatment of ASCVD.

## Conclusion

In summary, we combined molecular, biochemical and computational approaches to determine the binding site of apoA-I in DSC1 and to find small molecules that promote HDL biogenesis by inhibiting apoA-I-DSC1 interactions. Rutin, acarbose and docetaxel have been identified in this study and all of the three compounds have previously been shown to increase HDL *in vivo*, validating the usefulness of targeting apoA-I-DSC1 interactions to raise HDL. In addition to chemical compounds, antibodies and peptides blocking apoA-I-DSC1 interactions may also be developed as novel HDL biogenic therapies. The identification of docetaxel is particularly exciting as it is not only a potent promoter of HDL biogenesis, also a suppressor of VSMC proliferation. Cholesterol accumulation and VSMC proliferation in the arterial wall are biochemical hallmarks of atherosclerosis, therefore the dual action of docetaxel may exert synergistically atheroprotective effects. The high therapeutic potential warrants future studies to investigate if docetaxel prevents and/or reduce atherosclerosis in animal models.

## Data Availability

The raw data supporting the conclusions of this article will be made available by the authors, without undue reservation.

## Abbreviations

ABCA1, ATP binding cassette A1; AIBS, apoA-I binding site; apoA-I, apolipoprotein A-I; ASCVD, atherosclerotic cardiovascular diseases; DSC1, desmocollin 1; EC, extracellular cadherin; EC50, half-maximal effective concentration; EGFP, enhanced green fluorescent protein; FDA, United States Food and Drug Administration; HASMCs, human aortic smooth muscle cells; HDL-C, high density lipoprotein-cholesterol; HSFs, human skin fibroblasts; LDL, low density lipoprotein; OD, optical density; PDGF, platelet-derived growth factor; PM, plasma membrane; SRB, sulforhodamine B; TG, triglyceride; VSMCs, vascular smooth muscle cells.
