# Supplementary material for: Identification of Docetaxel as a Potential Drug to Promote HDL Biogenesis
Source: Front Pharmacol. 2021 May 21;12:679456. doi: 10.3389/fphar.2021.679456 (PMC8176524; doi:10.3389/fphar.2021.679456)
Supplement: Supplementary file 1 [file Presentation1.pdf]

Supplementary Material

1 Major Resources Table

1.1 Antibodies

Target antigen	Vendor or Source	Catalog #	Working concentration
ApoA-I	Meridian Life Science	K45252G	Western blot (1:1000)
EGFP	Clontech	632569	Western blot (1:1000)
Actin	Santa Cruz Biotechnology	sc-1616	Western blot (1:1000)
ABCA1	Millipore Sigma	MAB10005	Western blot (1:1000)
DSC1	Novus Biologicals	NBP1-88099	Western blot (1:200)
Tubulin	Abcam	ab7291	Western blot (1:5000)

1.2 Transfected Constructs

Plasmid Name	Sequence	Source / Reference
pDSC1b-EGFP	Full length human DSC1b followed by EGFP tag	Choi HY *et al.*, 2018
pDSC1bΔ(447-466)-EGFP	Deletion of DSC1b residues 447-466 from pDSC1b-EGFP	This paper
pDSC1bΔ(447-486)-EGFP	Deletion of DSC1b residues 447-486 from pDSC1b-EGFP	This paper
pDSC1bΔ(447-506)-EGFP	Deletion of DSC1b residues 447-506 from pDSC1b-EGFP	This paper
pDSC1bΔ(447-526)-EGFP	Deletion of DSC1b residues 447-526 from pDSC1b-EGFP	This paper
pDSC1bΔ(447-546)-EGFP	Deletion of DSC1b residues 447-546 from pDSC1b-EGFP	This paper

1.3 Cultured Cells

Name	Vendor or Source	Sex (F, M, or unknown)
Primary human skin fibroblasts	Obtained from a forearm skin biopsy in a healthy subject	M
HEK293	ATCC	Unknown
Human aortic smooth muscle cells	Thermo Fisher Scientific	Unknown
THP-1 monocytes	ATCC	M

1.4 Drugs

Name	Vendor or Source	Catalog #
Acarbose	Selleckchem	S1271
Rutin	Selleckchem	S2350
Docetaxel	Enamine	BRC0139

2 Supplementary Figures

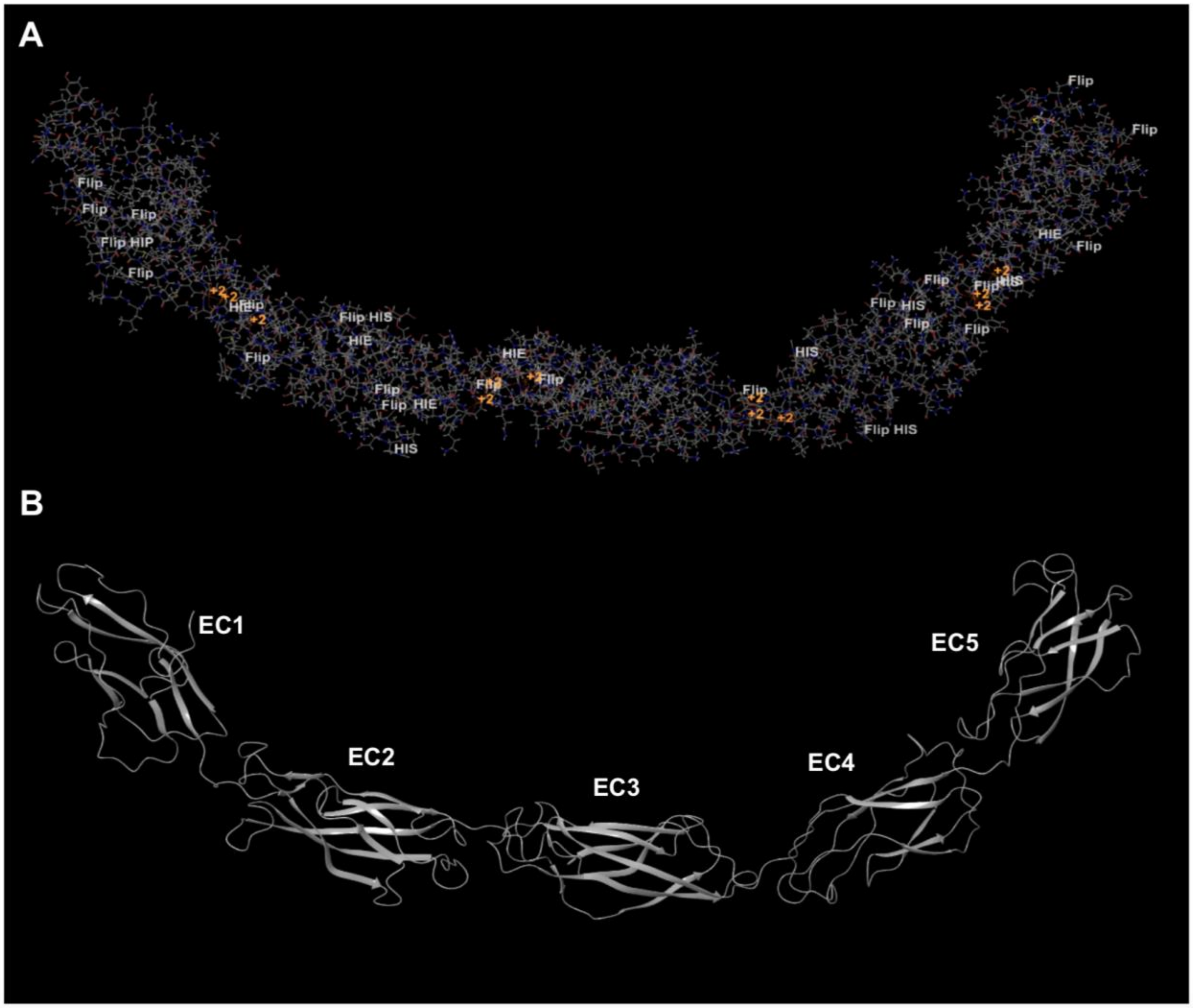

Supplementary Figure S1. Ball & Stick (A) and ribbon (B) models of DSC1.
A crystal structure of the human DSC1 ectodomain, 5IRY was optimized for structure-based drug design using the Protein Preparation Wizard implemented in Maestro. Some of the amino acid residues corrected by the Wizard are shown in (**A**): HIS, histidine; HIE, histidine with hydrogen on the epsilon nitrogen; HIP, histidine with hydrogens on both nitrogens; Flip, flip the terminal amide group of Asn or Gln; Flip HIS, flip the histidine ring; +2 denotes calcium ion. The five tandemly repeated extracellular cadherin domains (EC1-EC5) of DSC1 are shown in (**B**).

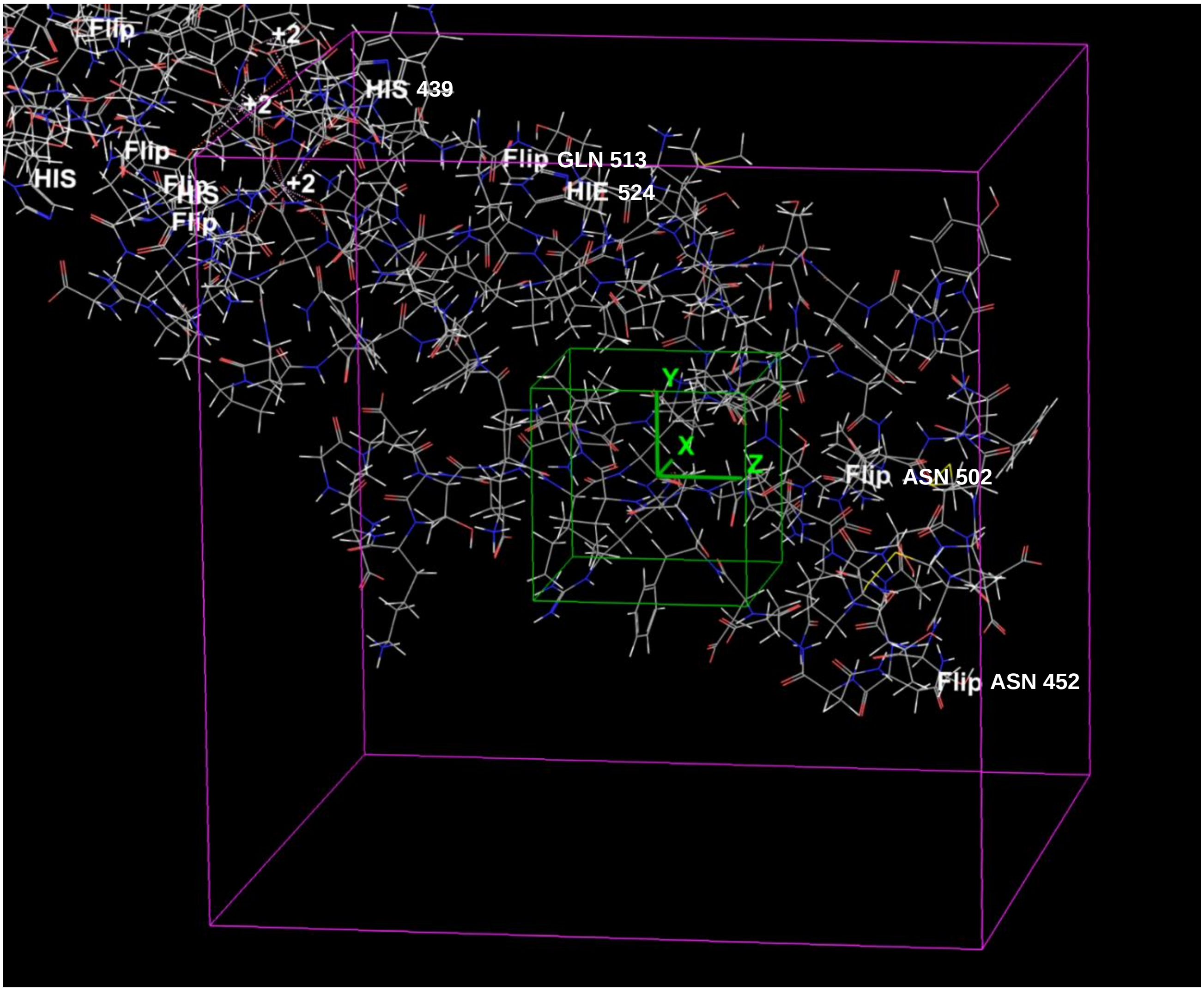

Supplementary Figure S2. Receptor grid generation.
The outer, purple enclosing box defines the volume in which the grid potentials were calculated. All atoms of a ligand must be located within the purple box. The inner, green center box defines the volume that the center of a ligand explores during the site-point search. Acceptable positions for the center of a ligand must lie within the green box. Amino acid residues that were corrected by the Protein Preparation Wizard and located within the purple box are labelled with their residue numbers. Calcium ions denoted as +2 are not included in the purple box. HIS: histidine; HIE: histidine with hydrogen on the epsilon nitrogen; Flip: flip the terminal amide group of Asn or Gln residue.

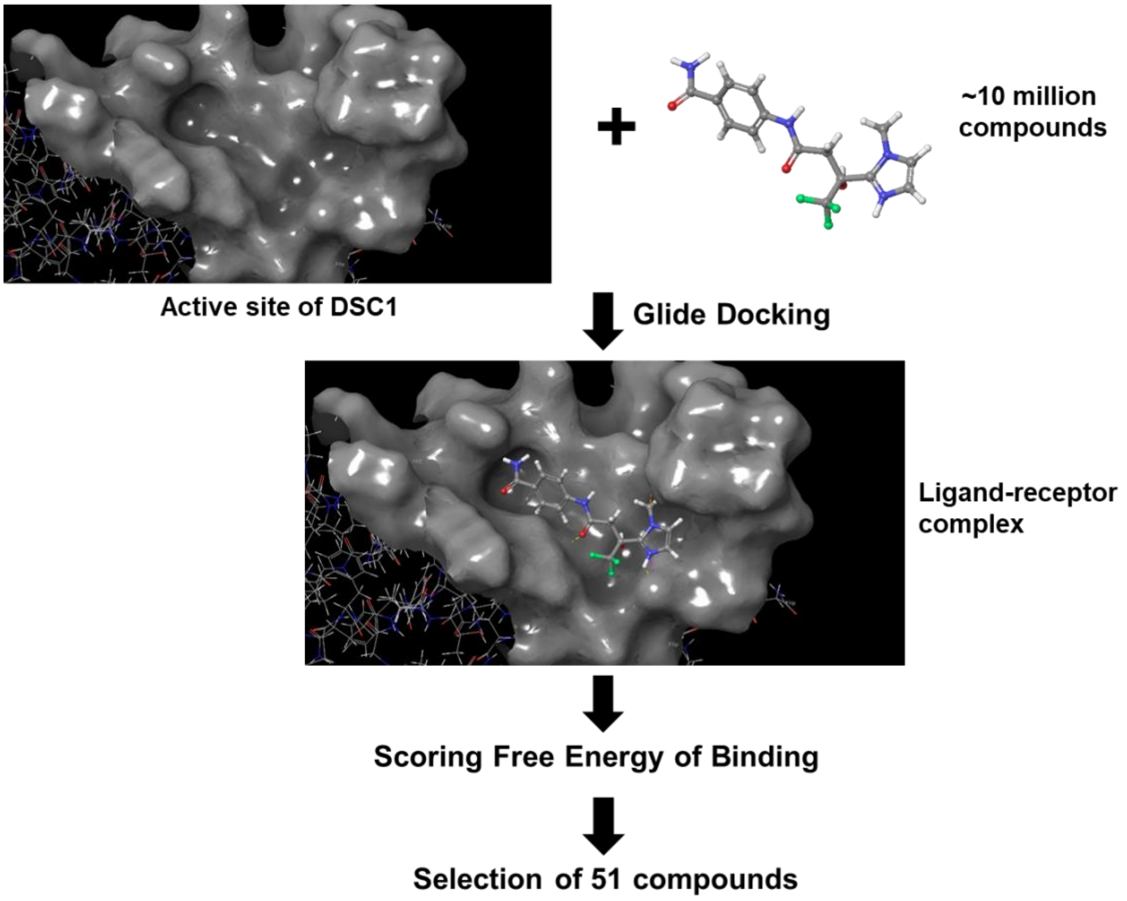

Supplementary Figure S3. Schematic diagram showing the work-flow of DSC1 active site structure-based virtual screening of ligands.

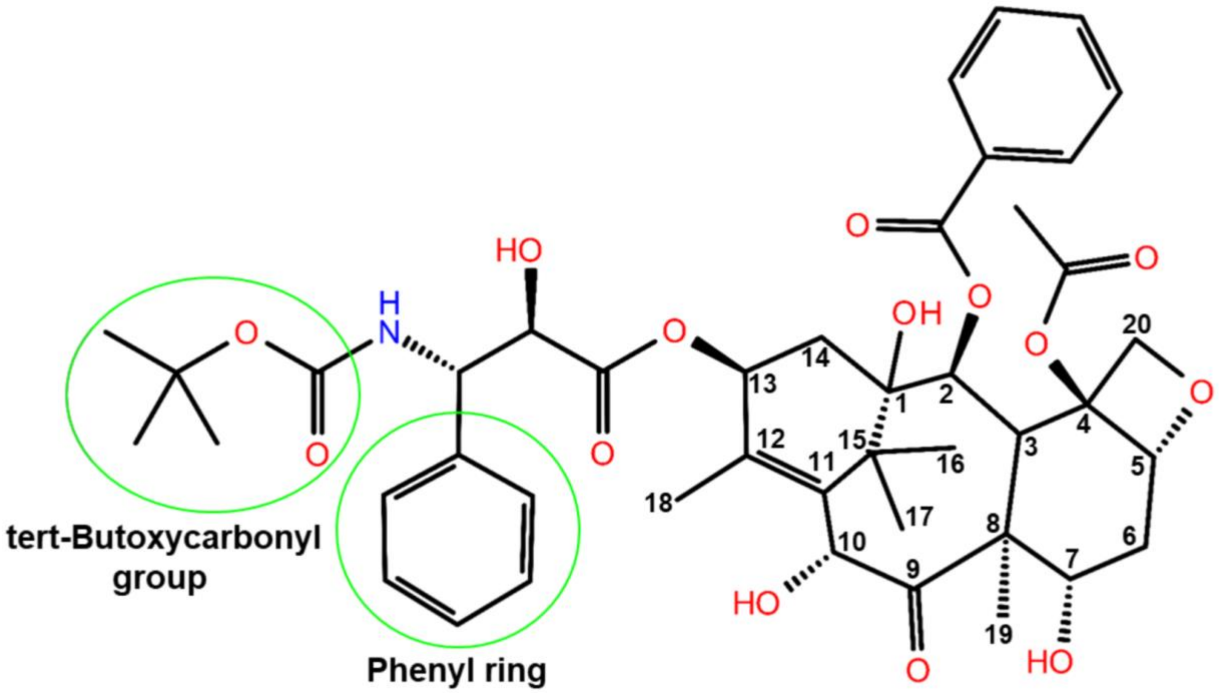

Supplementary Figure S4. The chemical structure of docetaxel with the number of carbon atoms in the taxane ring.
